# lncRNA AFAP1-AS1 promotes triple negative breast cancer cell proliferation and invasion via targeting miR-145 to regulate MTH1 expression

**DOI:** 10.1038/s41598-020-64713-x

**Published:** 2020-05-06

**Authors:** Xiaohui Zhang, Yidong Zhou, Feng Mao, Yan Lin, Songjie Shen, Qiang Sun

**Affiliations:** Department of Breast Surgery, Peking Union Medical College Hospital, Peking Union Medical College &Chinese Academy of Medical Sciences (CAMS), Beijing, China

**Keywords:** Breast cancer, Breast cancer

## Abstract

The actin fiber-associated protein 1-antisense RNA1 (AFAP1-AS1) is upregulated in various cancers and associated with cancer proliferation and metastasis. Several cancer-related pathways have been linked to up-expression of this long non-coding (lnc)RNA, but the underlying mechanisms are yet unknown. In triple negative breast cancer (TNBC), AFAP1-AS1 expression is also significantly overexpressed compared to that in other subtypes of breast cancer from the TCGA dataset. In this study, we performed bioinformatic RNAhybrid analyses and identified that miR-145 is a potential target of AFAP1-AS1 and able to reduce MutT homolog-1 (MTH1) expression. Thus, this study investigated the oncogenic activity of AFAP1-AS1 in TNBC cells and the underlying mechanisms that are yet poorly understood. The results showed that miR-145 expression was low, whereas AFAP1-AS1 and MTH1 expression was high in TNBC cells and that miR-145 mimics reduced TNBC cell proliferation and invasion, whereas miR-145 knockdown exerted the opposite activity in TNBC cells. Moreover, knockdown of AFAP1-AS1 reduced tumor cell proliferation and invasion, but miR-145 co-transfection rescued tumor cell viability and colony formation ability. The dual luciferase reporter assay showed that AFAP1-AS1 could directly target miR-145, while miR-145 could directly target MTH1. After knockdown of ATF6, AFAP1-AS1 was reduced along with AFAP1-AS1 promoter activity. This study revealed that AFAP1-AS1 could promote TNBC cell proliferation and invasion via regulation of MTH1 expression through targeting of miR-145.

## Introduction

Long non-coding RNAs (lncRNAs) are naturally occurring non-coding RNA 200 nucleotides or more in length that play important roles in the regulation of different biological processes of tumors^1,2^. The actin fiber-associated protein 1-antisense RNA1 (AFAP1-AS1), a newly discovered lncRNA, is the antisense of the actin filament-associated protein 1 (AFAP1), which is a Src-binding partner that functions as an adaptor protein by linking Src family members and/or other signaling proteins to actin filament^3^. AFAP1-AS1 expression is upregulated in various human cancers and associated with larger tumor size and advanced stages of esophageal squamous carcinoma cells^4–6^, lung cancer^7^, colorectal cancer^8^, and others for tumor progression^9–11^. In breast cancer, AFAP1-AS1 expression is dysregulated^12^ and associated with poor prognosis and progression of breast cancer phenotypes^13–15^. However, knockdown of AFAP1-AS1 expression was able to reduce expression of Ki-67 and matrix metalloproteinase but increase Bax expression, thereby suppressing tumor growth^14,16,17^, although the specific targets of AFAP1-AS1 and the related signaling pathways in cancer development and progression remain to be determined. Moreover, microRNAs are a class of non-coding RNAs up to 24 nucleotides in length that function to regulate the transcription and translation of protein-coding genes^18^. miR-145 has been reported to play an important role in the regulation of cancer cell growth, invasion, and metastasis^19,20^, and miR-145 overexpression was able to inhibit cancer cell growth by downregulating MAP3K1 in lung cancer^21^ and the PAK4-dependent pathway in colon cancer^22^. In breast cancer, alteration of miR-145 expression has also been frequently reported^23–26^, but only a few studies have investigated its upstream signaling pathways (e.g., MTH1, an enzyme, is capable of hydrolyzing the oxidized dNTPs and NTP, such as 8-oxo-dGTP and 2-OH-dATP, to form and prevent their incorporation into the nucleus and mitochondrial DNA to limit reactive oxygen species [ROS] production for induction of cell damage)^27,28^. Cancer cells generate a high level of MTH1 to reduce the harmful ROS effect, thereby escaping apoptosis and surviving^29–32^. A previous study reported that miR-145 can reduce MutT homolog-1 (MTH1) expression in lung adenocarcinoma and contribute to a significant inhibition of cell proliferation, indicating that miR-145 plays an inhibitory role in lung adenocarcinoma cell through suppression of MTH1 expression. The relationship between lncRNA and miRNA is believed to involve the function of lncRNAs as competitive RNAs (ceRNAs) for miRNAs (i.e., as miRNA sponges) to reduce the regulatory effects of mRNAs^33^. The preliminary data obtained in the present study via bioinformatic RNAhybrid^34^ analysis show that AFAP1-AS1 could target miR-145 expression after our bioinformatic RNAhybrid^34^ analysis. Thus, we hypothesized that AFAP1-AS1 overexpression could promote triple negative breast cancer (TNBC) cell proliferation and invasion through competitive binding to miR-145 to, in turn, upregulate MTH1 expression and TNBC cell phenotypes *in vitro*. We therefore, first assessed expression of AFAP1-AS1, miR-145, and MTH1 in normal breast cells and different breast cancer cell lines and then investigated the differential effects of miR-145 and AFAP1-AS1 on the regulation of breast cancer cell viability and invasion *in vitro* and *in vivo*. Next, we explored and confirmed their interactions in breast cancer cells.

## Materials and methods

### Cell culture

Breast cancer MDA-MB-231, MDA-MB-468, MDA-MB-435S, and HCC1937 cell lines and a normal mammary gland epithelial MCF-10A cell line were originally obtained from American Type Culture Collection (Manassas, VA, USA). MDA-MB-231 cells were cultured in Dulbecco’s modified Eagle’s medium (DMEM), while MDA-MB-468, MDA-MB-435S, and HCC1937 cells were cultured in Roswell Park Memorial Institute medium-1640 (RPMI-1640) supplemented with 10% fetal bovine serum (FBS) and 1% penicillin–streptomycin. MCF-10A cells were cultured in DMEM/F12 (1:1) containing horse serum (5%), penicillin-streptomycin (1%), insulin (10 μg/ml), epidermal growth factor (20 ng/ml), choleramycin (100 ng/ml), and hydrocortisone (0.5 μg/ml) in an incubator with 5% CO_2_ (Thermo Forma, Waltham, MA, USA) at 37 °C. All cell culture reagents were purchased from Gibco Laboratories (Grand Island, NY, USA).

### RNA isolation and quantitative real-time polymerase chain reaction (qRT-PCR)

Total RNA was isolated from cultured cells using the TRIzol Reagent (Invitrogen, Carlsbad, CA, USA) and reversely transcribed into cDNA using the high-capacity cDNA RT kit (Applied Biosystems, Foster City, CA, USA) according to the manufacturers’ instructions. For qPCR, these cDNA samples were amplified with the SYBR® Green PCR mix (Applied Biosystems) for levels of AFAP1-AS1 and miR-145 expression. The data were normalized to levels of β-actin and U6, respectively. The qPCR primers were purchased from RiboBio (Guangzhou, China), and the sequences are listed in Table 1.

**Table 1:** Primer sequences.

Gene name	Sequence
miR-145-5p for RT	5′-GTCGTATCCAGTGCAGGGTCCGAGGTGCACTGGATACGACAGGGATTC-3′
U6 for RT	5′-GTCGTATCCAGTGCAGGGTCCGAGGTGCACTGGATACGACAGGGATTC-3′
miR-145-5p	5′-TGCGGGTCCAGTTTTCCCAGGA-3′
	5′-CCAGTGCAGGGTCCGAGGT-3′
U6	5′-TGCGGGTGCTCGCTTCGGCAGC-3′
	5′-CCAGTGCAGGGTCCGAGGT-3′
AFAP1-AS1	5′-AATGGTGGTAGGAGGGAGGA-3′
	5′-CACACAGGGGAATGAAGAGG-3′
MTH1	5′-CTCAGCGAGTTCTCCTGG-3′
	5′-GGAGTGGAAACCAGTAGCTGTC-3′
β-actin	5′-CGTGACATTAAGGAGAAGCTG-3′
	5′-CTAGAAGCATTTGCGGTGGAC3-3′

### Luciferase reporter assay

To predict the target gene of AFAP1-AS1, we first performed a bioinformatics analysis. We identified potential genes and focused on miR-145 based on the RNAhybrid results. We then constructed vectors carrying the wild-type or mutated miR145 3′-untranslated region (3′-UTR), which are referred to as pmirGLO/AFAP1-AS1-3′UTR and pmirGLO/AFAP1-AS1-3′UTR Mut, respectively (the detailed construction protocol is described in the supplementary information). For the Luciferase reporter assay, MDA-MB-231 cells were seeded into 6-well plates at a density of 1 × 10^5^/well and grown in 2 mL DMEM for 24 h to reach 70%–80% confluency. The medium was then replaced with 1 mL DMEM without antibodies, and the cells were transfected with each luciferase reporter gene (20 pmol) diluted in 50 μL Opti-MEM and 4 μL Lipofectamine 2000 (Invitrogen) for 24 h. The dual luciferase assay was performed using a Dual luciferase reporter gene detection kit (Cat. #RG009, Biyuntian, Jiangsu, China) according to the manufacturer’s instructions on a GloMax machine (Promega, Madison, WI, USA). The data were normalized to Renilla luciferase activity.

### Cell viability assay

The viability of MDA-MB-231 cells was evaluated using the 3-(4,5-dimethylthiazol-2-yl)-2,5-diphenyltetrazolium bromide (MTT) assay. Specifically, cells were seeded into 96-well plates at a density of 5 × 10^3^/well and incubated overnight in 100 µL DMEM before transfection with miR-145 mimics or a negative control of miR-145 mimics, ASO-NC, ASO-miR-145, pSilencer-NC plus ASO-NC, pshR-AFAP1-AS1 plus ASO-NC, or pshR-AFAP1-AS1 plus ASO-miR-145 for 48 h. Before the end of each assay, 10 µL (5 mg/mL) of the MTT reagent (Sigma-Aldrich, USA) was added into a final volume of 100 µL DMEM and incubated for additional 4 h. After that, the culture medium was replaced with 100 μL dimethyl sulfoxide (DMSO), and the absorbance of each cell culture solution was measured using a microplate reader (Thermo Scientific, USA) at 570 nm. The experiments were performed in triplicate and repeated at least three times.

### Cell colony formation assay

After transfections, cells were reseeded into 12-well plates at a density of 200/well in 2 mL of complete growth medium and incubated for 2 weeks at 37 °C. The growth medium was replaced every 3 days. At the end of the experiments, cells were washed with ice-cold PBS twice and fixed with fresh-made 4% paraformaldehyde at 4 °C for 30 min. Next, cells were washed with PBS three times and stained with 0.1% crystal violet, and cell colonies with 50 cells or more were counted under an inverted microscope (Olympus, Japan). The experiment was repeated at least three times.

### Wound healing assay

MDA-MB-231 cells were seeded into 6-well plates at a density of 3 × 10^5^ cells/well and grown overnight. On the next day, the cells were transfected with miR-145 mimics or negative control, pshR-AFAP1-AS1 or pSilencer-NC, and negative control or pshR-AFAP1-AS1 plus ASO-miR-145 for 48 h. After cell monolayers reached 95%–98% confluency, a cell wound was created using a 200-μL sterile plastic tip, and then the cells were washed three times with PBS. The cells were further cultured in serum-free medium at 37 °C for 48 h and imaged under a phase-contrast microscope. The experiment was repeated at least three times.

### Transwell invasion assay

Tumor cell invasion capacity was assessed using a Transwell chamber (Millipore, Billerica, USA) with the filter precoated with 25 μL Matrigel (BD Biosciences, Franklin Lakes, NJ, USA). In brief, cells were seeded into the upper chamber with 200 μL serum-free medium at a density of 1 × 10^5^ cells/well, and the bottom chambers were filled with 500 μL DMEM supplemented with 20% FBS. After culture for 72 h, cells on the upper filter surface were removed using a cotton swab, while cells that had invaded the bottom side of the filter were fixed with a mixture of methanol and glacial acetic acid (a ratio of 3:1) for 30 min at room temperature and stained with 0.1% crystal violet for 15 min. The numbers of invading cells in three randomly selected fields on each filter were counted under a light microscope (Olympus, Japan). The assay was repeated at least three times.

### Western blot

Whole lysates of MDA-MB-231 cells were harvested in RIPA buffer (Sigma-Aldrich, USA) supplemented with a protease inhibitor cocktail (Thermo Fisher Scientific, USA) and quantified using the BCA protein assay kit (CWBIO, Beijing, China). Sodium dodecyl sulfate-polyacrylamide gel electrophoresis (SDS-PAGE) electrophoresis was used to separate proteins, and samples were transferred onto polyvinylidene fluoride (PVDF) membranes (Millipore, Billerica, MA, USA). The membranes were then blocked for 1 h at room temperature with a blocking buffer (5% skim milk in PBS) and further incubated with the primary antibodies at 4 °C overnight. These primary antibodies were a mouse anti-MTH1 and mouse anti-ATF6 antibodies (Tianjin Biotechnology Co., Ltd. Tianjin, China). On the next day, the membranes were washed with PBS-Tween 20 (PBS-T) three times and then incubated with secondary antibody (Tianjin Biotechnology Co., Ltd. Tianjin, China) for 1 h at room temperature. After washing with PBS-T three times, the protein bands were visualized using an enhanced chemiluminescence kit (Thermo Scientific, USA). GAPDH protein was used as a control. The assay was repeated at least three times.

### *In vivo* nude mouse tumor cell xenograft assay

The animal study protocol was approved by the Institutional Animal Care and Use Committee (IACUC) of the Peking Union Medical College Hospital (Beijing, China) and followed the Guidelines of the Care and Use of Laboratory Animals issued by the Chinese Council on Animal Research. Female Balb/c nude mice (4 weeks of age) were purchased from the Institute of Laboratory Animal Science, Chinese Academy of Medical Sciences (Beijing, China) and maintained in a specific pathogen-free (SPF) “barrier” facility. The mice were housed under controlled temperature and humidity and alternating 12-hour light and dark cycles. The mice received SPF mouse chow and sterile water ad libitum. The mice were randomly divided into 5 groups and each group contained 5 mice. MDA-MB-231 cells transfected with different genes (e.g., miR-145 mimics or negative control, pSilencer-NC or pshR-AFAP1-AS1 or pshR-AFAP1-AS1 plus ASO-miR-145) were grown, and 5 × 10^7^/mL cell suspensions were prepared in 100 μL PBS and subcutaneously injected into the back of each mouse on the left side. Mouse weight and tumor formation and size were monitored daily and recorded, and the tumor volumes were calculated from measurements of the longest (L) and shortest (S) tumor dimensions taken every 3 days using the formula: V = (L × S^2^)/2. After 3–5 weeks, the nude mice were anesthetized with intraperitoneal injection of 80 mg/kg of ketamine and 10 mg/kg of xylazine according to standard procedures and photographed. Finally, mice were euthanized by cervical dislocation and the tumor xenografts were removed and weighed.

### Statistical analysis

All statistical analyses were performed using SPSS version 15.0 software (SPSS, Chicago, IL, USA). All of our experiments were repeated three times, and the data are presented as mean ± standard error. Student’s *t* test was used for comparisons between two groups, and one-way analysis of variance with the Bonferroni post-test was used for comparisons among three or more groups. A two-side value of *P* < 0.05 was considered statistically significant.

## Results

### Differential expression of miR-145, AFAP1-AS1, and MTH1 in normal breast cells and different breast cancer cell lines

In this study, we first analyzed AFAP1-AS1 expression in TNBC and found that AFAP1-AS1 expression was significantly higher in TNBC than in other subtypes of breast cancer using TCGA dataset (Figure S1) We also found that expression levels of miR-145 and MTH1 in TNBC were obviously lower and higher than those in luminal breast cancer, respectively (Figures S2 and S3).

**Figure 1:**
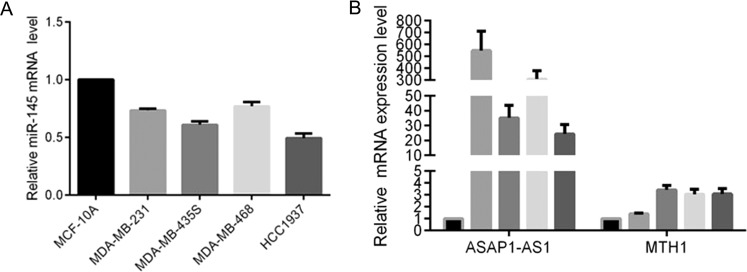
Differential expression of miR-145, AFAP1-AS1, and MTH1 in breast cancer cell lines. (Normal breast cells and different breast cancer cell lines were subjected to qRT-PCR analysis of (**A**) miR-145 expression as well as (**B**) AFAP1-AS1 and MTH1 expression.

We then assayed their expression in breast cancer cell lines and found that the expression level of miR-145 was lower in breast cancer cells compared with that in normal mammary epithelial MCF-10A cells (Fig. 1A). In contrast, the expression levels of AFAP1-AS1 and MTH1 were higher in breast cancer cells compared with those in MCF-10A cells (Fig. 1B).

### Differential effects of miR-145 and AFAP1-AS1 on regulation of breast cancer cell viability and invasion

**Figure 2:**
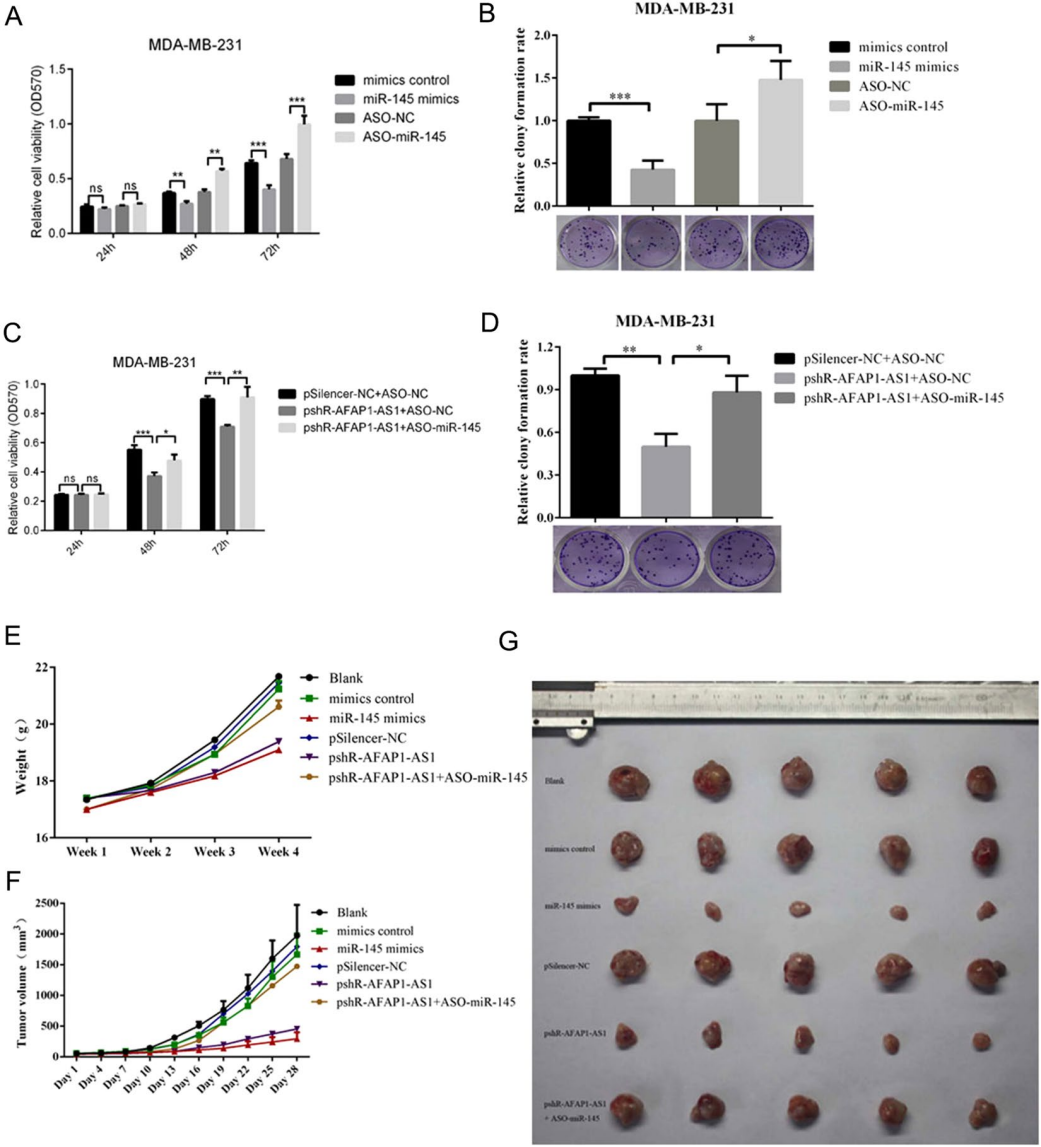
Differential effects of miR-145 and AFAP1-AS1 on the regulation of breast cancer cell viability and colony formation *in vitro* and tumor formation *in vivo*. (**A**) Cell viability assay. MDA-MB-231 cells were transiently transfected with miR-145 mimics, negative control, ASO-miR-145, or ASO-NC for 48 h, and cell viability was analyzed by MTT assay. (**B**) Colony formation assay. MDA-MB-231 cells were transiently transfected with miR-145 mimics, negative control, ASO-miR-145, or ASO-NC for 48 h and subjected to a colony formation assay. The graph shows the summarized data from the assay. (**C**) Cell viability assay. MDA-MB-231 cells were transiently transfected with pSilencer-NC plus ASO-NC, pshR-AFAP1-AS1 plus ASO-NC, or pshR-AFAR1-AS1 plus ASO-miR-145 for 48 h, and cell viability was assessed by the MTT assay. (**D**) Colony formation assay. MDA-MB-231 cells were transiently transfected with pSilencer-NC plus ASO-NC, pshR-AFAP1-AS1 plus ASO-NC, or pshR-AFAR1-AS1 plus ASO-miR-145 for 48 h before analysis with a colony formation assay. The graph shows the summarized data from the assay. (**E**) Changes in mouse body weight in nude mouse tumor cell xenograft model. (**F**) Changes in tumor volume in nude mouse tumor cell xenograft model. (**G**) Photographs of tumor xenografts. **p < 0.05 and ***p < 0.01.

Furthermore, we found that transfection with miR-145 mimics reduced MDA-MB-231 cell viability and colony formation capacity, whereas knockdown of miR-145 using ASO-miR-145 had the opposite effects on breast cancer cell viability and colony formation (Fig. 2A,B). Moreover, knockdown of AFAP1-AS1 expression by pSilence-AFAP1-AS1 transfection reduced the viability and colony formation capacity of MDA-MB-231 cells (Fig. 2C,D), whereas miR-145 co-transfection rescued tumor cell viability and colony formation ability (Fig. 2C,D). The same tumor growth result was observed in the nude mouse xenograft assay. Specifically, miR-145 mimics inhibited the formation and size of MDA-MB-231 cell-derived xenografts, whereas knockdown of AFAP1-AS1 inhibited MDA-MB-231 tumor growth in mice (Fig. 2E–G).

### Differential effects of miR-145 and AFAP1-AS1 on regulation of breast cancer cell wound healing and invasion *in vitro*

**Figure 3:**
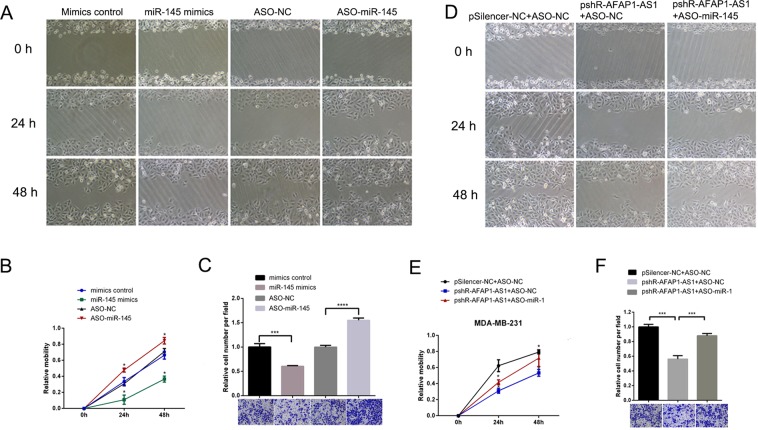
Differential effects of miR-145 and AFAP1-AS1 on the regulation of breast cancer cell wound healing and invasion *in vitro*. (**A**) Wound healing assay. MDA-MB-231 cells were transiently transfected with miR-145 mimics, negative control, ASO-miR-145, or ASO-NC for 48 h before use in a wound healing assay. (**B**) Quantified data from a. (**C**) Transwell invasion assay. MDA-MB-231 cells were transiently transfected with pSilencer-NC plus ASO-NC, pshR-AFAP1-AS1 plus ASO-NC, or pshR-AFAR1-AS1 plus ASO-miR-145 for 48 h before use in a Transwell invasion assay. (**D**) Wound healing assay. MDA-MB-231 cells were transiently transfected with pSilencer-NC plus ASO-NC, pshR-AFAP1-AS1 plus ASO-NC, or pshR-AFAR1-AS1 plus ASO-miR-145 for 48 h before use in a wound healing assay. (**E**) Quantified data from d. (**F**) Transwell invasion assay. MDA-MB-231 cells were transiently transfected with pSilencer-NC plus ASO-NC, pshR-AFAP1-AS1 plus ASO-NC, or pshR-AFAR1-AS1 plus ASO-miR-145 for 48 h for use in a Transwell invasion assay. The graph shows the summarized data from the assay. **p < 0.05 and ***p < 0.01.

We found that miR-145 mimics reduced MDA-MB-231 cell wound healing and invasion *in vitro*, whereas knockdown of miR-145 using ASO-miR-145 had the opposite effects on breast cancer cell wound healing and invasion *in vitro* (Fig. 3A–C). Moreover, knockdown of AFAP1-AS1 expression by pSilence-AFAP1-AS1 also reduced the wound healing and invasion capacities of MDA-MB-231 cells *in vitro* (Fig. 3D–F), whereas ASO-miR-145 rescued tumor cell viability and colony formation ability (Fig. 3D–F).

### Interaction of miR-145 with AFAP1-AS1 and miR-145 with MTH1 in breast cancer cells

**Figure 4:**
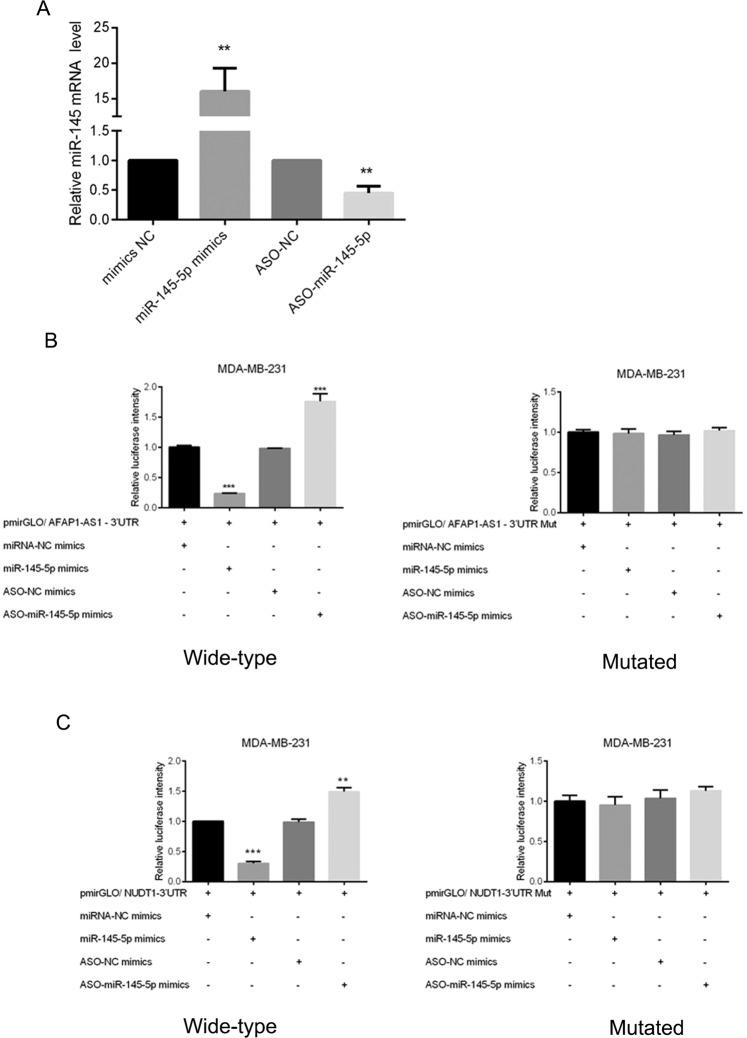
Interaction of AFAP1-AS1 with miR-145 and miR-145 with MTH1 in breast cancer cells. (**A**) qRT-PCR. MDA-MB-231 cells were transiently transfected with miR-145 mimics, negative control, ASO-miR-145, or ASO-NC for 48 h and subjected to qRT-PCR analysis of miR-145 expression. (**B**) Luciferase assay. MDA-MB-231 cells were transiently transfected with pmirGLO/AFAP1-AS1 3′-UTR, miR-145 mimics, negative control, ASO-miR-145, or ASO-NC for 48 h and subjected to Luciferase assay. The right panel shows the results of the Luciferase assay with mutated pmirGLO/AFAP1-AS1 3′-UTR transfection. (**C**) Luciferase assay. MDA-MB-231 cells were transiently transfected with pmirGLO/NUDT1 3′-UTR, miR-145 mimics, negative control, ASO-miR-145, or ASO-NC for 48 h and subjected to Luciferase assay. The right panel shows the results of the Luciferase assay with mutated pmirGLO/NUDT1 3′-UTR transfection. **p < 0.05 and ***p < 0.01.

We found that miR-145 mimics induced miR-145 expression in MDA-MB-231 cells, whereas miR-145-ASO knocked down miR-145 expression in MDA-MB-231 cells (Fig. 4A). We then successfully constructed vectors carrying wild-type or mutated AFAP1-AS1 3′-UTRs (named pmirGLO/AFAP1-AS1-3′-UTR and pmirGLO/AFAP1-AS1-3′UTR Mut, respectively; Figure S4), the wild-type and mutated pmiRGLO-ATF6-3′-UTR plasmids (Figure S5**)**, and the wild-type and mutated pmiGLO/NUDT1-3′-UTR and pmiGLO/ NUDT1-3′-UTR plasmids (Figure S6**)**. The results of dual luciferase reporter assays showed that AFAP1-AS1 could directly target miR-145 (Fig. 4B), while miR-145 could directly target MTH1 (Fig. 4C). In contrast, the mutated vectors resulted in no changes in luciferase activity (Fig. 4B,C).

**Figure 5:**
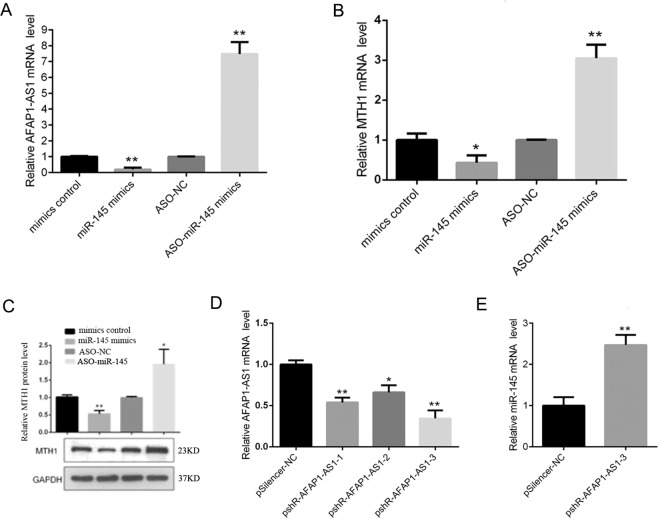
Interaction of miR-145 with AFAP1-AS1 and miR-145 with MTH1 in breast cancer cells. (**A**) qRT-PCR. MDA-MB-231 cells were transiently transfected with miR-145 mimics, negative control, ASO-miR-145, or ASO-NC for 48 h and subjected to qRT-PCR analysis of AFAP1-AS1 expression. (**B**) qRT-PCR. MDA-MB-231 cells were transiently transfected with miR-145 mimics, negative control, ASO-miR-145, or ASO-NC for 48 h and subjected to qRT-PCR analysis of MTH1 expression. **(C**) Western blot. MDA-MB-231 cells were transiently transfected with miR-145 mimics, negative control, ASO-miR-145, or ASO-NC for 48 h and subjected to Western blot analysis of MTH1 expression. (**D**) qRT-PCR. MDA-MB-231 cells were transiently transfected with three different AFAP1-AS1 siRNAs and subjected to qRT-PCR analysis of AFAP1-AS1 expression. (**E**) qRT-PCR. MDA-MB-231 cells were transiently transfected with AFAP1-AS1-3 siRNA and then subjected to qRT-PCR analysis of miR-145 expression. **p < 0.05 and ***p < 0.01.

In addition, we explored their interaction and expression in MDA-MB-231 cells and found that miR-145 overexpression reduced the levels of AFAP1-AS1 and MTH1 in MDA-MB-231 cells, whereas knockdown of miR-145 expression enhanced their levels in MDA-MB-231 cells (Fig. 5A–C). After knockdown of AFAP1-AS1 expression (Fig. 5D), miR-145 expression was upregulated (Fig. 5E).

### Interaction of miR-145 with ATF6 and ATF6 feedback with AFAP1-AS1 in breast cancer cells

**Figure 6:**
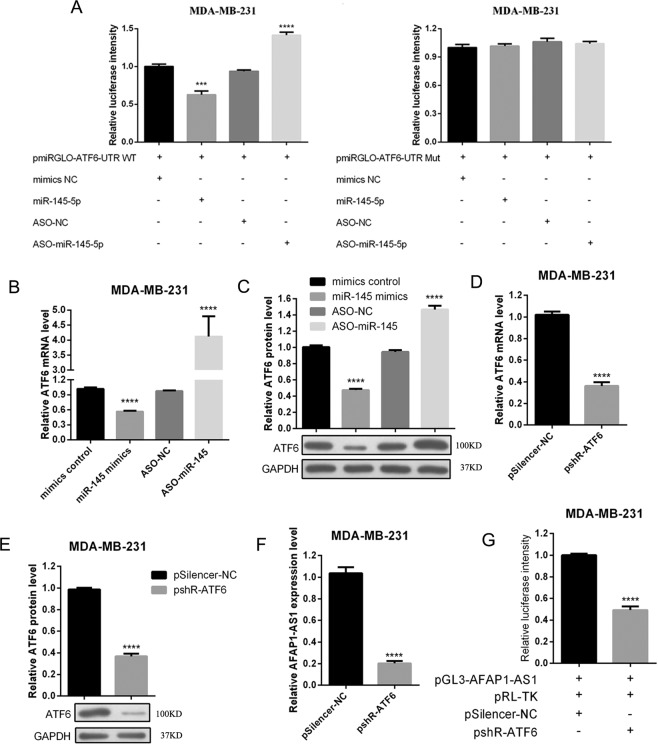
Interaction of miR-145 with ATF6 and ATF6 feedback with AFAP1-AS1 in breast cancer cells. (**A**) Dual fluorescent reporter assay. MDA-MB-231 cells were transiently transfected with pmirGLO/ATF6 3′-UTR, miR-145 mimics, negative control, ASO-miR-145, or ASO-NC for 48 h and subjected to Luciferase assay. The right panel shows the results of the Luciferase assay with mutated pmirGLO/ATF6 3′-UTR transfection. (**B**) qRT-PCR. MDA-MB-231 cells were transiently transfected with miR-145 mimics, negative control, ASO-miR-145, or ASO-NC for 48 h and subjected to qRT-PCR analysis of ATF6 expression. (**C**) Western blot. MDA-MB-231 cells were transiently transfected with miR-145 mimics, negative control, ASO-miR-145, or ASO-NC for 48 h and subjected to Western blot analysis of ATF6 protein expression. (**D**) qRT-PCR. MDA-MB-231 cells were transiently transfected with ATF6 siRNA or negative control for 48 h and subjected to qRT-PCR analysis of ATF6 expression. (**E**) Western blot. MDA-MB-231 cells were transiently transfected with ATF6 siRNA or negative control for 48 h and subjected to Western blot analysis of ATF6 protein expression. (**F**) qRT-PCR. MDA-MB-231 cells were transiently transfected with ATF6 siRNA or negative control for 48 h and subjected to qRT-PCR analysis of AFAP1-AS1 expression. (**G**) Luciferase assay. MDA-MB-231 cells were transiently transfected with pGL3-AFAP1-AS1, pRL-TK, pSilencer-NC, and/or pshR-ATF6 for 48 h and subjected to Luciferase assay to measure AFAP1-AS1 luciferase activity. ***p < 0.05 and ***p < 0.001.

We next explored how these genes interact in breast cancer cells by constructing wild-type and mutated pmiGLO/ATF6-3′-UTR and pmiGLO/ATF6-3′-UTR plasmids (Figure S5) and performed luciferase reporter assays. Our results showed that hsa-miR-145-5p also was able to directly bind to ATF6-3′-UTR, but this targeting effect disappeared when the seed sequences were mutated (Fig. 6A**)**. miR-145 expression or knockdown also changed the levels of AFT6 mRNA and protein in MDA-MB-231 cells (Fig. 6B,C). Moreover, knockdown of ATF6 expression effectively reduced the levels of ATF6 mRNA and protein (Fig. 6D,E) as well as the level of AFAP1-AS1 in breast cancer cells (Fig. 6F). In addition, ATF6 could directly bind to the promoter fragment of AFAP1-AS1 (Figures S7 and S8). After ATF6 was knocked down, the promoter activity of AFAP1-AS1 was reduced (Fig. 6G).

## Discussion

TNBC is characterized by a high recurrence rate, high potential metastasis, and poor treatment response and prognosis^35–37^. Research to better understand the molecular mechanisms and to support the development of novel molecular targeting therapeutic strategies for TNBC is a significant and hot topic in the field. In this study, we first analyzed TCGA dataset and found that AFAP1-AS1 expression was significantly higher in TNBC vs. other subtypes of breast cancer, while the expression levels of miR-145 and MTH1 were obviously lower and higher in TNBC than those in luminal breast cancer, respectively. Our *in vitro* and *in vivo* experiments further showed that AFAP1-AS1 expression was up-regulated in breast cancer cells and promoted TNBC cell proliferation and invasion *in vitro* as well as tumor formation and growth in nude mice. These data are consistent with previous studies showing that AFAP1-AS1 expression is elevated in breast cancer and promotes tumor proliferation^14,38^. These results indicate that AFAP1-AS1-miR145-MTH1 is an important CeRNA network in TNBC. Furthermore, AFAP1-AS1 has been demonstrated to be associated with poor prognosis in some cancer patients^39,40^. Based on this, we analyzed the relationships between AFAP1-AS1, miR-145, MTH1 and disease-free survival (DFS) and overall survival (OS) in TNBC patients from TCGA dataset and found no significant relationship (Figure S9). The possible reason is that the number of cases in TCGA is small and more cases are needed for verification. On the other hand, the prognosis is related to multiple factors, and the corresponding regulatory mechanisms require further research.

Altered expression of different miRNAs occurs and has been reported in breast cancer, but which miRNA interacts with AFAP1-AS1 is unclear. We performed RNAhybrid bioinformatics analysis and found that miR-145 could be a target gene of AFAP1-AS1. In the present study, dual luciferase reporter assays showed that AFAP1-AS1 could directly target miR-145, which confirmed the results of the bioinformatics analysis. For the effect on cell proliferation, we observed that knockdown of AFAP1-AS1 alone could reduce cell proliferation and invasion, but co-transfection of miR-145 rescued tumor cell viability and colony formation ability. These results are consistent with the previous report that miR-145 is one of nine miRNAs in a miRNA signature that may serve as a potential diagnostic marker for breast cancer^24^. A previous genetic association study showed that miR-145 single nucleotide polymorphisms (SNPs) are associated with breast cancer susceptibility^25^, while downregulation of miR-145 can be used to predict the risk of postmenopausal breast cancer^26^. Furthermore, upregulated miR-145 expression through demethylation of the mi*R145* promoter inhibits breast cancer cell migration and invasion^23^.

Furthermore, we also observed a positive association between AFAP1-AS1 and MTH1 and found that AFAP1-AS1 can increase MTH1 expression through downregulation of miR-145 in breast cancer cells *in vitro*. These results were consistent with a previous report that miR-145 expression can reduce MTH1 expression to suppress cancer cell proliferation^31^. Because cancer cells grow fast, they produce a large amount of ROS and are in a state of high oxidative stress. MTH1 can convert oxidized nucleoside triphosphates to nucleoside monophosphates, thereby preventing these oxidized nucleoside triphosphates from being incorporated into DNA to reduce cell death. Therefore, cancer cells have increased MTH1 expression to avoid ROS-induced cell damage, while normal cells have low expression levels of MTH1 because intracellular ROS levels are low^27–30^. Consistently, we found that the expression levels of MTH1 were higher in breast cancer cells compared with those in MCF-10A cells in this study.

In addition, our current study also revealed that ATF6 is a target gene of miR-145, or in other words, miR-145 inhibits ATF6 expression in TNBC cells, while ATF6 can directly bind to the AFAP1-AS promoter. Thus, knockdown of ATF6 expression led to reduced AFAP1-AS1 promoter activity and expression in TNBC cells. Taken together, these findings reveal a positive feedback among these three genes; i.e., ATF6 increases AFAP1-AS1 promoter activity and expression, which leads to a decrease in the miR-145 level and an increase in ATF6 expression in TNBC cells. However, the importance of ATF6 in breast cancer requires further study, because to date, there has been no study reporting the role of ATF6 in breast cancer.

## Conclusions

In summary, our current study established a ceRNA-based regulatory network in TNBC cell proliferation and invasion. We also demonstrated the important role of the lncRNA AFAP1-AS1 in the promotion of TNBC proliferation via regulation of MTH1 expression through targeting of miR-145 in breast cancer cells. The identified ATF6/AFAP1-AS1/miR-145 feedback mechanism could play an important role in the regulation of TNBC proliferation and invasion, and the interaction of these factors represents a novel therapeutic target in the treatment of TNBC patients that warrants further investigation.

## Supplementary information

Supplementary Information 1.

Supplementary Information 2.
