# Supplementary material for: lncRNA AFAP1-AS1 promotes triple negative breast cancer cell proliferation and invasion via targeting miR-145 to regulate MTH1 expression
Source: Sci Rep. 2020 May 6;10:7662. doi: 10.1038/s41598-020-64713-x (PMC7203232; doi:10.1038/s41598-020-64713-x)
Supplement: Supplementary file 1 — Supplementary Information 1. [file 41598_2020_64713_MOESM1_ESM.doc]

**lncRNA AFAP1-AS1 promotes triple negative breast cancer cell proliferation and invasion via targeting miR-145 to regulate MTH1 expression**

**Running title:** Oncogenic AFAP1-AS1 in TNBC cells

Xiaohui Zhang, Yidong Zhou, Feng Mao, Yan Lin, Songjie Shen, Qiang Sun

Department of Breast Surgery, Peking Union Medical College Hospital, Peking Union Medical College &Chinese Academy of Medical Sciences (CAMS), Beijing, China.

**Corresponding author**

Qiang Sun

Department of Breast Surgery, Peking Union Medical College Hospital, Peking Union Medical College &Chinese Academy of Medical Sciences (CAMS), No.1 Shuaifuyuan Street, Beijing 100730, China.

E-mail: sunqpumch@163.com

Tel: +86-13811669255

**Supplementary Information**

**
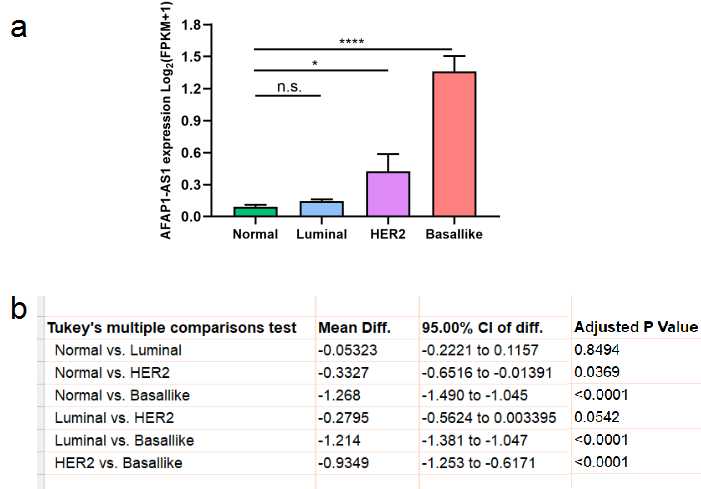
**

**Figure S1.** (a) AFAP1-AS1 expression in normal breast tissues and luminal, HER2 and basal-like breast cancers. (b) Table illustration of the comparison results between different groups.

**
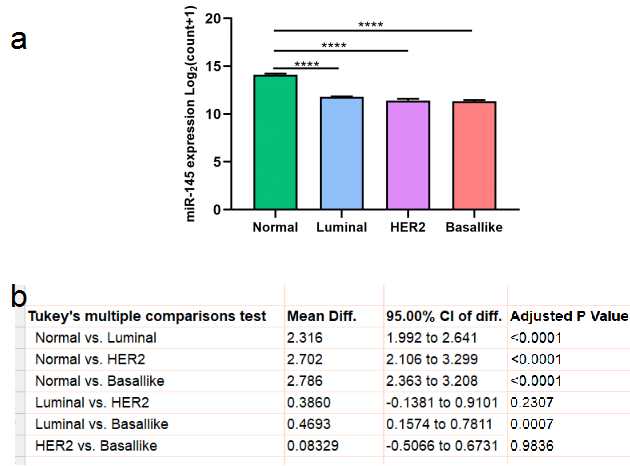
**

**Figure S2.** (a) miR-145 expression in normal breast tissues and luminal, HER2 and basal-like breast cancers. (b) Table illustration of the comparison results between different groups.

**
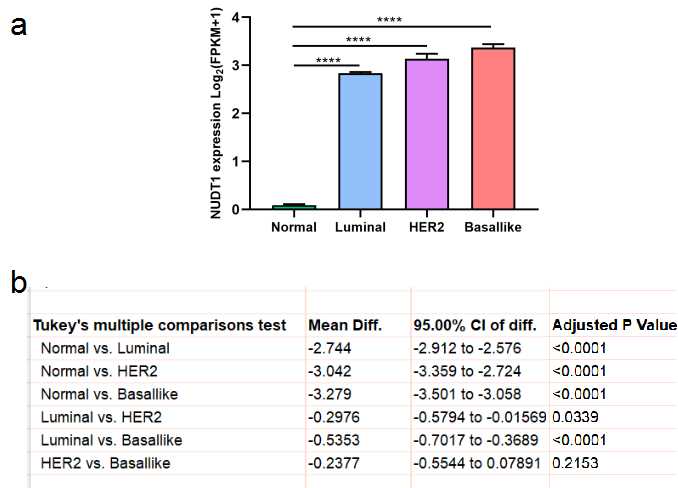
**

**Figure S3.** (a) MTH1 (NUDT1) expression in normal breast tissues and luminal, HER2 and basal-like breast cancers. (b) Table illustration of the comparison results between different groups.

**Construction of wild-type and mutated pmirGLO/AFAP1-AS1-3'-UTR and pmirGLO/AFAP1-AS1-3'-UTR vectors**

AFAP1-AS1: Location NC_000004.12, Reference GRCh38.p13 (GCF_000001405.39). Primary Assembly (7754090…7778928).

DNA fragments containing wild-type and mutated AFAP1-AS1-3'-UTRs were synthesized, annealed to form double-stranded DNA, and then subcloned into pmirGLO (Promega, Madison, WI, USA).

The DNA sequences for the wild-type AFAP1-AS1-3'-UTR were PmeI-5'-AAACTAGCGGCCGCTAGTTCAGAAGAGACACGAGGACTGGAGCTCAGCTTGCATCTCTACTGGATGATTAAGAATAAAAACACTGGAT-3'-Xbal and Xbal-5'-CTAGATCCAGTGTTTTTATTCTTAATCATCCAGTAGAGATGCAAGCTGAGCTCCAGTCCTCGTGTCTCTTCTGAACTAGCGGCCGCTAGTTT-3'-PmeI, whereas those for the mutated AFAP1-AS1-3'-UTR were PmeI-5'-AAACTAGCGGCCGCTAGTTCAGAAGAGACACGAGGTGACCTGCTCAGCTTGCATCTCTTGACCTTGATTAAGAATAAAAACTGACCTT-3'-Xbal and Xbal-5'-CTAGAAGGTCAGTTTTTATTCTTAATCAAGGTCAAGAGATGCAAGCTGAGCAGGTCACCTCGTGTCTCTTCTGAACTAGCGGCCGCTAGTTT-3'-PmeI. After amplification in *Escherichia coli*, these vectors were verified by DNA sequencing and used in our *in vitro* experiments.


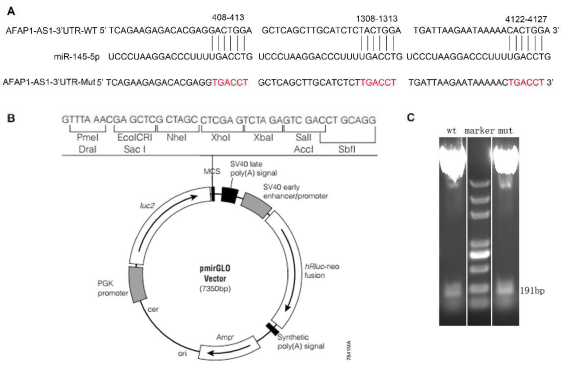


**Figure S4.** Construction of wild-type and mutated pmirGLO/AFAP1-AS1-3'UTR vectors. A, Wild-type and mutated AFAP1-AS1-3'UTR sites. B, The vector used for plasmid construction. C, Agarose gel image showing wild-type and mutated AFAP1-AS1-3'UTR fragments. wt, wild-type UTR; mut, mutant UTR.

**Construction of pSilencer2.1-U6/AFAP1-AS1 (abbreviated as pshR-AFAP1-AS1) vector**

DNA fragments containing three different AFAP1-AS1 shRNAs were synthesized, annealed to form double-stranded DNA, and then subcloned into pSilencer2.1-U6 (Promega). The DNA sequences for three different AFAP1-AS1 shRNAs were shR-AFAP1-AS1-1-top, 5'-GATCCGCTTCCTTCTCTACGTCTTCAAATCCTTCCTCGAGGAAGGATTTGAAGACGTAGAGAAGGAAGCTTTTTGA-3'; shR-AFAP1-AS1-1-bot, 5'-AGCTTCAAAAAGCTTCCTTCTCTACGTCTTCAAATCCTTCCTCGAGGAAGG ATTTGAAGACGTAGAGAAGGAAGCG-3'; shR-AFAP1-AS1-2-top. 5'-GATCCGCCCTGCCACTTGCTTGTCTGCATGTGTGCTCGAGCACACATGCAGACAAGCAAGTGGCAGGGCTTTTTGA-3'; shR-AFAP1-AS1-2-bot, 5'-AGCTTCAAAAAGCCCTGCCACTTGCTTGTCTGCATGTGTGCTCGAGCACACATGCAGACAAGCAAGTGGCAGGGCG-3'; shR-AFAP1-AS1-3-top, 5'-GATCCGTTCTGGGCTTCAATTTACAAGCAGTCAGCTCGAGCTGACTGCTTGTAAATTGAAGCCCAGAACTTTTTGA-3'; and shR-AFAP1-AS1-3-bot, 5'-AGCTTCAAAAAGTTCTGGGCTTCAATTTACAAGCAGTCAGCTCGAGCTGACTGCTTGTAAATTGAAGCCCAGAACG-3'.

**Construction of wild-type and mutated pmirGLO/ATF6-3**'**UTR vectors**

ATF6: Location NC_000001.11, Reference GRCh38.p13 (GCF_000001405.39). Primary Assembly (161766320…161964070).

DNA fragments containing wild-type and mutated ATF6-3'-UTRs were synthesized, annealed to form double-stranded DNA, and then subcloned into pmirGLO (Promega). The DNA sequences for the wild-type ATF6-3'-UTR wt-Top were PmeI-5'-AAACTAGCGGCCGCTAGT CCATCTATTTGGAAAGCACTGGAAT-3'-XbaI; ATF6-3'UTR wt-Bot, XbaI-5'-CTAGATTCCAGTGCTTTCCAAATAGATGGACTAGCGGCCGCTAGTTT-3'-PmeI; ATF6-3'UTR mut-Top, PmeI-5'-AAACTAGCGGCCGCTAGTCCATCTATTTGGAAAGCTGACCTAT-3'-XbaI; and ATF6-3'UTR mut-Bot, XbaI-5'-CTAGATAGGTCAGCTTTCCAAATAGATGGACTAGCGGCCGCTAGTTT-3'-PmeI.


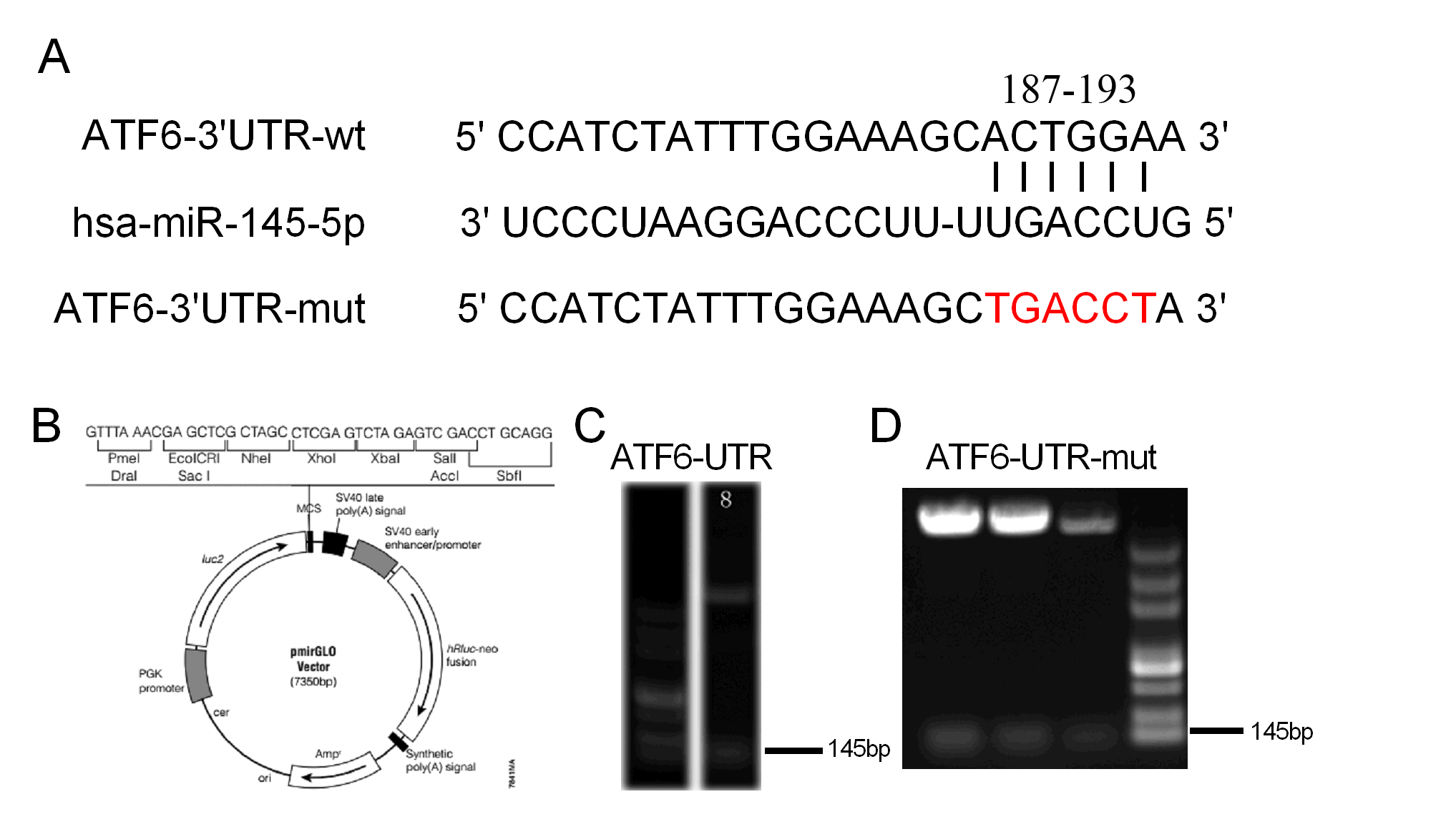


**Figure S5.** Construction of wild-type and mutated pmirGLO-ATF6-3'UTR vectors. A, The wild-type and mutated ATF6 3'-UTR sites. B, Vectors used for carrying ATF6-3'-UTR. C, Agarose gel image showing the wild-type ATF6-3'UTR. D, Mutated ATF6-3'UTR.

**Construction of wild-type and mutated pmirGLO/NUDT1-3**'-**UTR vectors**

NUDT1: Location NC_000007.14, Reference GRCh38.p13 (GCF_000001405.39). Primary Assembly (2242222…2251145).

DNA fragments containing wild-type and mutated NUDT1-3'-UTRs were synthesized, annealed to form double-stranded DNA, and then subcloned into pmirGLO (Promega). The DNA sequences for the wild-type NUDT1-3'-UTR wt-Top were

PmeI 5´AAACTAGCGGCCGCTAGTGTTTCATCTGGAATTAACTGGA T 3´ Xbal

NUDT1-3'UTR wt-Bot,

Xbal5´CTAGATCCAGTTAATTCCAGATGAAACACTAGCGGCCGCTAGTTT3´ PmeI

NUDT1-3'UTR mut-Top,

PmeI: 5´AAACTAGCGGCCGCTAGTGTTTCATCTGGAATTTTGACCT T 3´ Xbal

and NUDT1-3'UTR mut-Bot,

Xbal 5´CTAGAAGGTCAAAATTCCAGATGAAACACTAGCGGCCGCTAGTTT 3´ PmeI


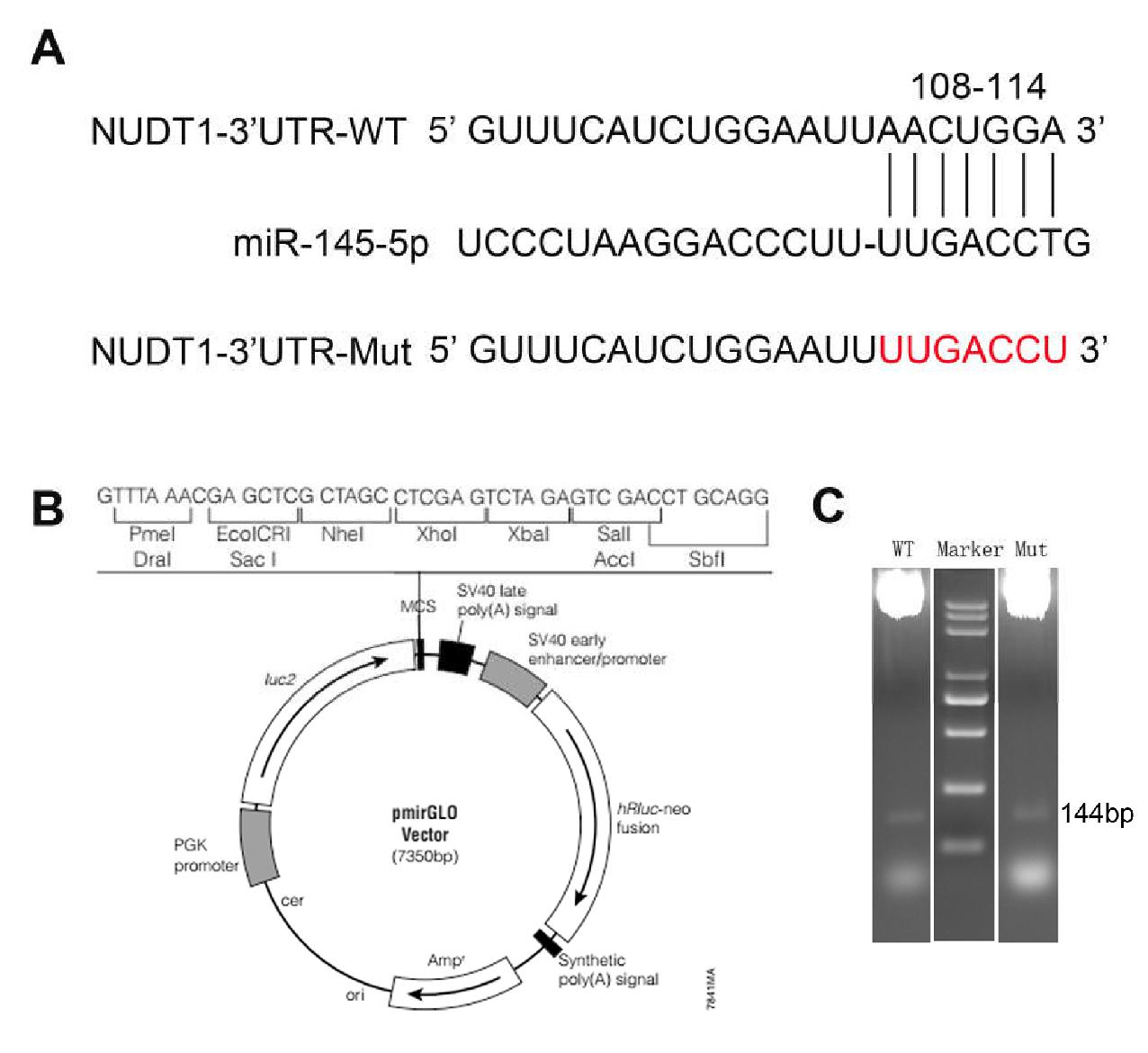


**Figure S6.** Construction of wild-type and mutated pmirGLO/NUDT1-3'UTR vectors. A, Wild-type and mutated NUDT1-3'-UTR sites. B, Vectors used to carry NUDT1-3'-UTR. C, Agarose gel image showing the wild-type and mutated NUDT1-3'-UTR.

**Construction of pGL3-AFAP1-AS1 vector**

The promoter region of AFAP1-AS1 (nucleotides -2000 to 0 bp) was chemically synthesized and cloned into the pGL3-Basic luciferase vector (Promega). The following AFAP1-AS1 promoter sequence was obtained from the NCBI:

5'-GGTACCCTGACCACCACAAACCCTTGCTATGATTGGTGAGCCACATGGTCTGGTTGCCCAAGGA (Restriction site of KpnI) ATTTGCTCGAGGTCAGAGACAG CGTCCATTCACTCCCCCAAAACTGTGAGGAGTCTTCCTTGGTGCACATCACTCCAGTAAGTGTCTTTGGGGAAGGTTAGGAGGAAATCTTTGTTATAAATTCATGCCAGGACCATGCCTAAGGGGCTATAACATCCCCCCGTATTCAAGGGCCTTCGGGTCTGCCACCTGGCTCAGGTCTGGGGATGGATTGATAAGGGCCCTAAGTTTCTCGGTGACTCAAGCAGGGTCTCTAATTCCCAGAGCCAGATAGGGCATCTTTGTTTCTCTGTTTGCTTTGTGCAGATCAAGGGAAGACCTTAGTGGCCTGTGTGGGGACCTCAATCCCAGAGACACCCTGATCCCTTCATCACCACTGACACTCTGTGGGTTCAACCATGATGATTATGGCTGAGCTCTTGTTTCCCATCCCAATACACAAGCCCACTTTGTCTCTGGAAATTAAGAGCTGCCATTATACCTATGGAATGCGAGAACCCAGTTTCAGTGGGGATGGCTCCTGTTCTCAGCTCTGAGTTGAAAAAGCAGTCATCATAGAGCTTTTCAAGAAAGATCTTGGAACCAGTGGACGCCTCCCAACACCTTAGCCAATGGAGTGTCCTCTGGGCCCTGCAAGTGCATGATGACACAGAGGGTGAGGCTGCAGCACATGACCACCAGACCCACGTGACACGCCCATCCCCCCCATGCCTGACGGTTTCCTCCCCATGTGCCCATGGGAGGCCTGCTTCACTTTGCAATCCGCCACAGCCAGAAGCCCAAGTCCTCTATATCCTCTCCATGACTTATAATTGAATGATTAGTCAAAGAAAATGGGACAGTCTCTAAAGCTGAAAAGATAACCCACAGAGAAAGGATTTCTGCTCGATTAAAGTCTTACGGGTGTCGTGGGATAAGTCACGGATCAGTACGTGCCCAATACTTGACGAGTGTAAAACAGACCTTGGGCCGGGTGCAGTGGCTCACGCCTGTAATCCCAGCACTTTGGGAGGCTGAGGCAGGCGGATCACCTGAGGTCCGGAGTTCGAGACCAGCCTGGCCAACATGGAGAAACCTGTCTCTACTACAAATACAAAATTAGCCGGGCGTGGTGGCAGGTGCCTGTAATCCAGCTACTCCAGAGGCTGAGGCAGGAGAATGGCTTGAAGCCGGGAGGCGGAGGTTGGTGTGAGCCGAGATCACGCCATTGCACTCCAGCCTGGGCAACAAGAGCGAAACTCCGTCTAAAAAAAGAAAAACAGACCTTGAATGCTCGGCCGGTGAGCTGACTCATGGAAAGCACCTGGAACAAAGCCTGGCACAGAGCGGACGCCGTGAGAGGATCCGCCGCTGCTGCTGCGGTTAAATGAGTGTTTCTGAAAAGCTTCCTGGGCCAAGCCAGTGCTAGACAGCAACAAGGCAGCAACAGGCAGCCAGCGCCTGCCCCCAAAGAGTTCCCAGTCTCTGGGAATGTGAAACAGGCTTAAAACCAGGGGTAATATCATAGGAAAAGTGCAAGGAAAGGATTCTCCTGATGGATTCATCCAGAGCGATGGGACCTGAAAGCATTGTGCATATTGATTGCAGAAGAAGCAGACCTGTGCAGGGAGAGAAGCATCAGAACACGCACCTGTGCAGGGAGATAAGCATCAGAACACGCACCTGTGCAGGAAGAGAGAGGCATTGGGACACACACCTGTGCAGGGAGAGAGGCATTAGAGAGGCAGGGAAGGAGGTGATATGGTTTGGCTGTATCCCCATCCAAATCTCAGCTTGAATTATATTTCCCAGAATTCCCATGCATTGTGGGAGGGACCCAGGGGAAGGTAATTGAATCATGGGGGCTGGTCTTCCCCGTTATTCTCGTGATAGTGAGTAAGTCTCACGATACCTGGAGGGTTTATCAAGGGTTCCTGCTTTTGCTGCTTCCTCGTTTTCTTTTGCTAGC-3' (Restriction site of NheI).


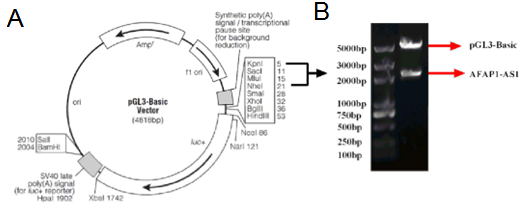


**Figure S7.** Construction of pGL3-AFAP1-AS1 vector. A, Plasmid used to subclone AFAP1-AS1 DNA fragment. B, Agarose gel image showing the AFAP1-AS1 DNA fragment.

**PCR analysis**

The primers were designed based on the binding sites of AFAP1-AS1 promoter sequences and ATF6, which were AFAP1-AS1-CHIP-F, 5'-GGGATGGCTCCTGTTCTCAG-3' and AFAP1-AS1-CHIP-R, 5'-CATGGGGAGGAAACCGTCAG-3'. The PCR protocol was set for a reaction system with 2 µL CHIP DNA, 5 µL 10 x PCR buffer (Mg^2+^-plus), 1 µL of 10 mM dNTP, 1 µL each of the forward and reverse primers (20 mM), 0.5 µL Taq polymerase (Takara, Dalian, China), and 39.5 µL sterile water. The mixture was then heated to 94°C for 4 min, followed by 30 cycles of 94°C for 45 s, 58°C for 45 s and 72°C for 1 min and a final step of 72°C for 10 min. The product was subjected to 2% agarose gel electrophoresis.


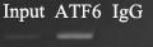


**Figure S8.** Agarose gel electrophoresis of the PCR product. The data show ATF6 binding to the AFAP1-AS1 promoter.


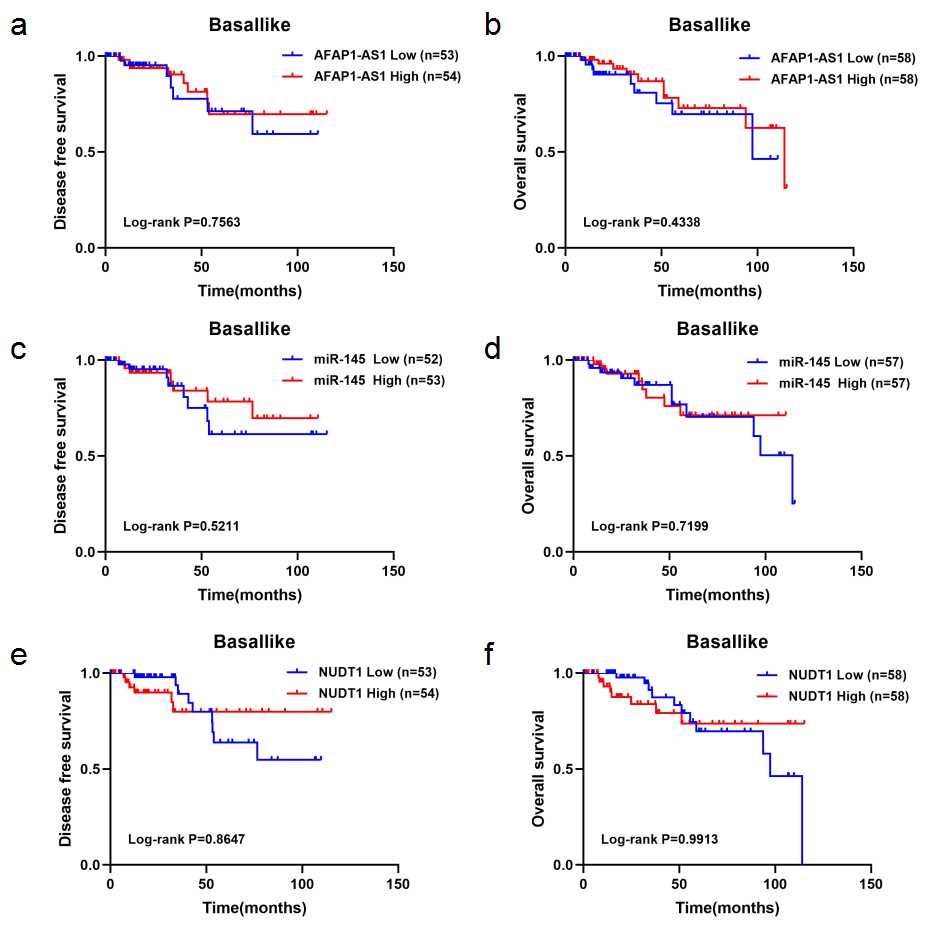


**Figure S9.** The relationships between (a-b) AFAP1-AS1, (c-d) miR-145, and (e-f) MTH1 (NUDT1) expression and disease-free survival (DFS) and overall survival (OS) in TNBC patients from TCGA dataset.
