# Supplementary material for: lncRNA AFAP1-AS1 promotes triple negative breast cancer cell proliferation and invasion via targeting miR-145 to regulate MTH1 expression
Source: Sci Rep. 2020 May 6;10:7662. doi: 10.1038/s41598-020-64713-x (PMC7203232; doi:10.1038/s41598-020-64713-x)

lncRNA AFAP1-AS1 promotes triple negative breast cancer cell proliferation and invasion via targeting miR-145 to regulate MTH1 expression

Running title: Oncogenic AFAP1-AS1 in TNBC cells

Xiaohui Zhang, Yidong Zhou, Feng Mao, Yan Lin, Songjie Shen, Qiang Sun

Department of Breast Surgery, Peking Union Medical College Hospital, Peking Union Medical College &Chinese Academy of Medical Sciences (CAMS), Beijing, China.

Corresponding authors

Qiang Sun

Department of Breast Surgery, Peking Union Medical College Hospital, Peking Union Medical College &Chinese Academy of Medical Sciences (CAMS), Beijing, China.

E-mail: sunqpumch@163.com

Tel: +86-13811669255

Supplementary information 2

Figure 5 GAPDH

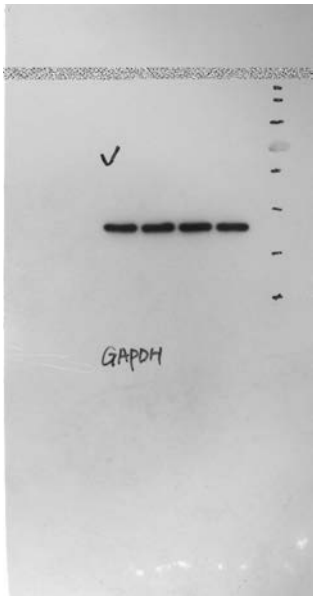

Figure 5 MTH1

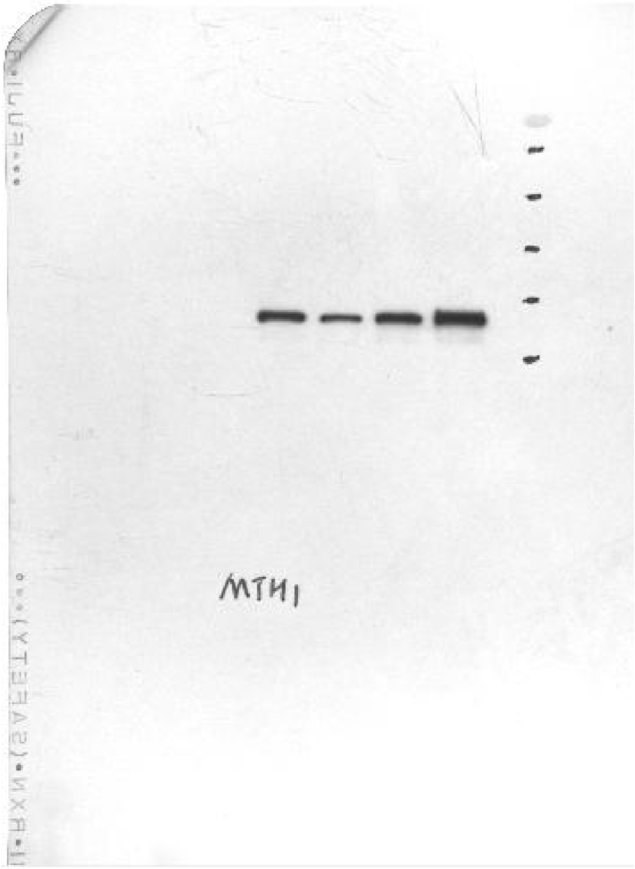

Figure 6 ATF6

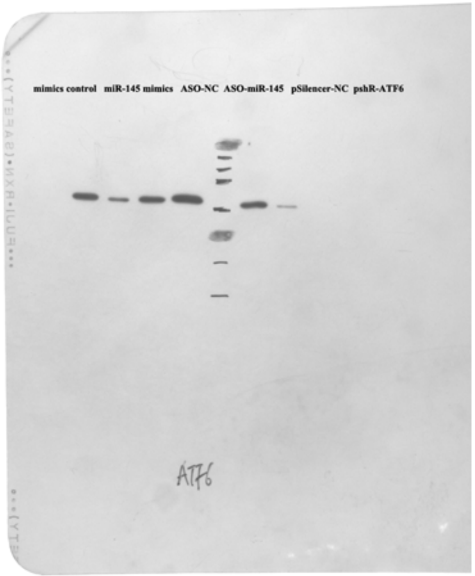

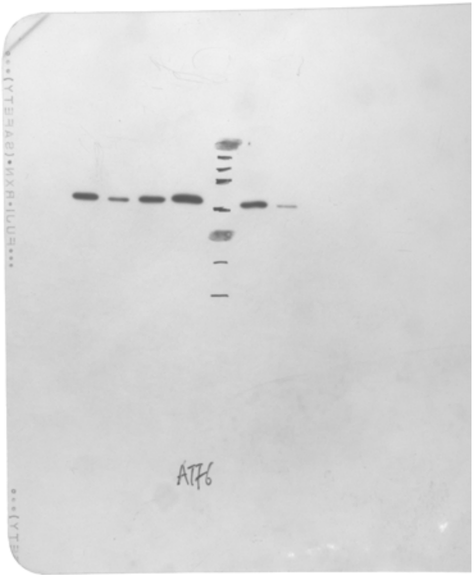

Figure 6 GAPDH

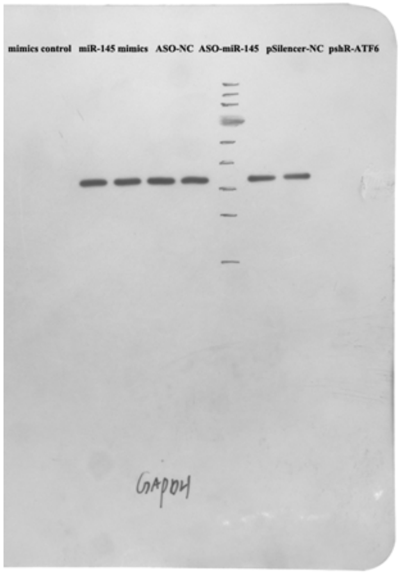

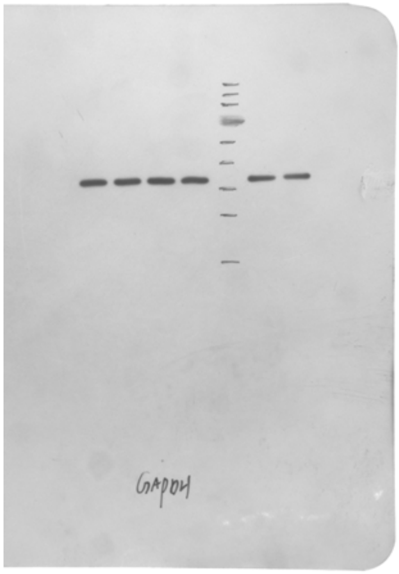

Supplement: Supplementary file 2 — Supplementary Information 2. [file 41598_2020_64713_MOESM2_ESM.pdf]
